# {4,4′,6,6′-Tetra­iodo-2,2′-[(2,2-dimethyl­propane-1,3-di­yl)bis­(nitrilo­methanylyl­idene)]diphenolato}nickel(II)

**DOI:** 10.1107/S1600536812024944

**Published:** 2012-06-16

**Authors:** Hadi Kargar, Reza Kia, Tayebeh Shakarami, Muhammad Nawaz Tahir

**Affiliations:** a Department of Chemistry, Payame Noor University, PO Box 19395-3697 Tehran, I. R. of IRAN; b Department of Chemistry, Science and Research Branch, Islamic Azad University, Tehran, Iran; c Structural Dynamics of (Bio)Chemical Systems, Max Planck Institute for Biophysical Chemistry, Am Fassberg 11, 37077, Göttingen, Germany; d Department of Physics, University of Sargodha, Punjab, Pakistan

## Abstract

The asymmetric unit of the title compound, [Ni(C_19_H_16_I_4_N_2_O_2_)], comprises half of a Schiff base complex. The Ni^II^ atom is located on a twofold rotation axis which also bis­ects the central C atom of the 2,2-dimethyl­propane group of the ligand. The geometry around the Ni^II^ atom is distorted square-planar, with a dihedral angle of 21.7 (3)° between the symmetry-related N/Ni/O coordination planes. The dihedral angle between the symmetry-related benzene rings is 27.9 (3)°. In the crystal, short inter­molecular I⋯I [3.8178 (9) and 3.9013 (10) Å] inter­actions are present.

## Related literature

For applications of Schiff bases in coordination chemistry, see: Granovski *et al.* (1993 ▶); Blower *et al.* (1998 ▶). For the related structures studied by our group, see: Kargar *et al.* (2012*a* ▶,*b* ▶,*c* ▶). For standard bond lengths, see: Allen *et al.* (1987 ▶). For van der Waals radii, see: Bondi (1964 ▶).
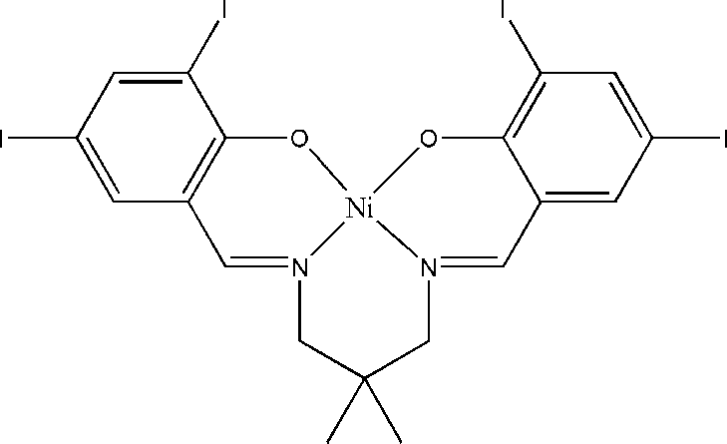


## Experimental

### 

#### Crystal data

[Ni(C_19_H_16_I_4_N_2_O_2_)]
*M* *_r_* = 870.65
Orthorhombic, 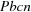

*a* = 16.682 (2) Å
*b* = 15.9978 (19) Å
*c* = 8.7920 (9) Å
*V* = 2346.4 (5) Å^3^
*Z* = 4
Mo *K*α radiation
μ = 6.11 mm^−1^
*T* = 291 K
0.21 × 0.15 × 0.11 mm

#### Data collection

Bruker SMART APEXII CCD area-detector diffractometer
Absorption correction: multi-scan (*SADABS*; Bruker, 2005 ▶) *T*_min_ = 0.360, *T*_max_ = 0.553
10345 measured reflections
2582 independent reflections
1615 reflections with *I* > 2σ(*I*)
*R*_int_ = 0.078

#### Refinement

*R*[*F* ^2^ > 2σ(*F* ^2^)] = 0.043
*wR*(*F* ^2^) = 0.096
*S* = 0.96
2582 reflections
129 parameters
H-atom parameters constrained
Δρ_max_ = 1.07 e Å^−3^
Δρ_min_ = −0.73 e Å^−3^

### 

Data collection: *APEX2* (Bruker, 2005 ▶); cell refinement: *SAINT* (Bruker, 2005 ▶); data reduction: *SAINT*; program(s) used to solve structure: *SHELXS97* (Sheldrick, 2008 ▶); program(s) used to refine structure: *SHELXL97* (Sheldrick, 2008 ▶); molecular graphics: *SHELXTL* (Sheldrick, 2008 ▶); software used to prepare material for publication: *SHELXTL* and *PLATON* (Spek, 2009 ▶).

## Supplementary Material

Crystal structure: contains datablock(s) global, I. DOI: 10.1107/S1600536812024944/su2444sup1.cif

Structure factors: contains datablock(s) I. DOI: 10.1107/S1600536812024944/su2444Isup2.hkl

Additional supplementary materials:  crystallographic information; 3D view; checkCIF report

## Figures and Tables

**Fig. 1.**
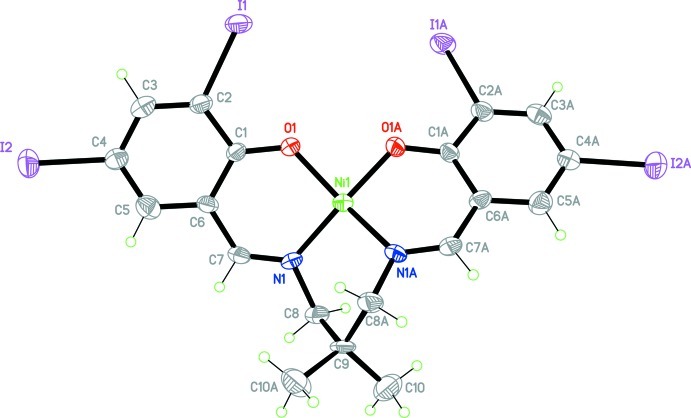
A view of the molecular structure of the title compound, showing 40% probability displacement ellipsoids and the atomic numbering [symmetry code for suffix A: -x + 1, y, -z+ 1/2].

**Fig. 2.**
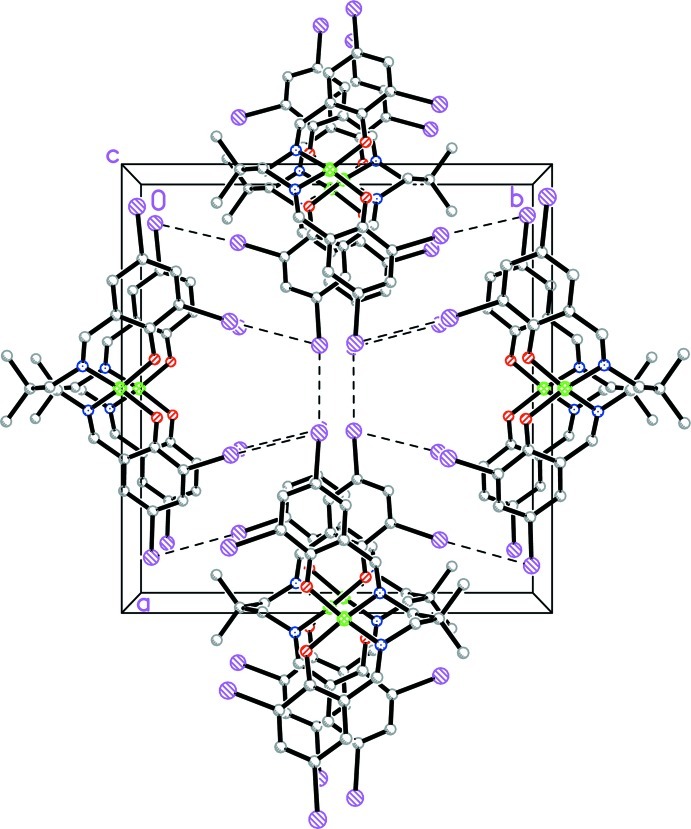
A view along the c axis of crystal packing of the title compound, showing the intermolecular I···I interactions (dashed lines).

## supplementary crystallographic information

### Comment

Schiff base complexes are one of the most important stereochemical models in transition metal coordination chemistry, with the ease of preparation and structural variations (Granovski *et al.*, 1993; Blower *et al.*, (1998). In continuation of our work on the crystal structure of Schiff base metal complexes (Kargar *et al.*, 2012*a*,*b*), we report herein on the crystal structure of the title compound, the nickel(II) complex of the Schiff base ligand 6,6'-(((2,2-dimethylpropane-1,3-diyl)bis(azanylylidene))bis(methanylylidene))bis(2,4-diiodophenol). The structure of the Zwitterion form of this ligand has been reported by us (Kargar *et al.*, 2012*c*).

The asymmetric unit of the title compound, Fig. 1, comprises half of a Schiff base complex. The Ni^II^ atom is located on a 2-fold rotation axis which also bisects the central C atom, C9, of the 2,2-dimethylpropane group in the ligand. The bond lengths (Allen *et al.*, 1987) and angles are within normal ranges and are comparable to those reported for the ligand (Kargar *et al.*, 2012*c*) and related structures (Kargar *et al.*, 2012*a*,*b*). The geometry around atom Ni1 is distorted square-planar which is supported by the N_2_O_2_ donor atoms of the coordinated Schiff base ligand. The dihedral angle between the benzene rings (C1-C6 and C1^i^-C6^i^; symmetry code: (i) -x+1, y, -z+1/2) is 27.9 (3)°, while that between the symmetry-related coordination planes, N1,Ni1,O1 and N1^i^,Ni1^i^,O1^i^ is 21.7 (3)°.

In the crystal, short intermolecular I1···I2^i^ [3.8178 (9)Å; symmetry code: (i) -x+3/2, -y-1/2, z+1/2] and I2···I2^ii^ [3.9013 (10)Å; symmetry code: (ii) -x+2, y, -z-1/2] interactions are present (Fig. 2). These interactions are shorter than the sum of the van der Waals radius of I atoms [3.96Å; Bondi, 1964].

### Experimental

The title compound was synthesized by adding 2 mmol of 6,6'-(((2,2-dimethylpropane-1,3-diyl)bis(azanylylidene))bis(methanylylidene)) bis(2,4-diiodophenol) to a solution of NiCl_2_. 6H_2_O (2.1 mmol) in ethanol (30 ml). The mixture was refluxed with stirring for 30 min. The resultant solution was filtered. Dark-red block-like crystals of the title compound, suitable for *X*-ray structure analysis, were recrystallized from ethanol by slow evaporation of the solvents at room temperature over several days.

### Refinement

The H-atoms were included in calculated positions and treated as riding atoms: C—H = 0.93, 0.96 and 0.97 Å for CH, CH_3_ and CH_2_ H-atoms, respectively, with U_iso_ (H) = k x U_eq_(C), where k = 1.5 for CH_3_ H-atoms, and = 1.2 for other H-atoms.

### Crystal data

**Table:**

[Ni(C_19_H_16_I_4_N_2_O_2_)]	*F*(000) = 1600
*M**_r_* = 870.65	*D*_x_ = 2.465 Mg m^−^^3^
Orthorhombic, *P**b**c**n*	Mo *K*α radiation, λ = 0.71073 Å
Hall symbol: -P 2n 2ab	Cell parameters from 2540 reflections
*a* = 16.682 (2) Å	θ = 2.5–27.4°
*b* = 15.9978 (19) Å	µ = 6.11 mm^−^^1^
*c* = 8.7920 (9) Å	*T* = 291 K
*V* = 2346.4 (5) Å^3^	Block, dark-red
*Z* = 4	0.21 × 0.15 × 0.11 mm

### Data collection

**Table:**

Bruker SMART APEXII CCD area-detector diffractometer	2582 independent reflections
Radiation source: fine-focus sealed tube	1615 reflections with *I* > 2σ(*I*)
Graphite monochromator	*R*_int_ = 0.078
φ and ω scans	θ_max_ = 27.1°, θ_min_ = 1.8°
Absorption correction: multi-scan (*SADABS*; Bruker, 2005)	*h* = −21→21
*T*_min_ = 0.360, *T*_max_ = 0.553	*k* = −20→18
10345 measured reflections	*l* = −11→9

### Refinement

**Table:**

Refinement on *F*^2^	Primary atom site location: structure-invariant direct methods
Least-squares matrix: full	Secondary atom site location: difference Fourier map
*R*[*F*^2^ > 2σ(*F*^2^)] = 0.043	Hydrogen site location: inferred from neighbouring sites
*wR*(*F*^2^) = 0.096	H-atom parameters constrained
*S* = 0.96	*w* = 1/[σ^2^(*F*_o_^2^) + (0.0314*P*)^2^] where *P* = (*F*_o_^2^ + 2*F*_c_^2^)/3
2582 reflections	(Δ/σ)_max_ = 0.001
129 parameters	Δρ_max_ = 1.07 e Å^−^^3^
0 restraints	Δρ_min_ = −0.73 e Å^−^^3^

### Special details

**Table:**

Geometry. All esds (except the esd in the dihedral angle between two l.s. planes) are estimated using the full covariance matrix. The cell esds are taken into account individually in the estimation of esds in distances, angles and torsion angles; correlations between esds in cell parameters are only used when they are defined by crystal symmetry. An approximate (isotropic) treatment of cell esds is used for estimating esds involving l.s. planes.
Refinement. Refinement of F^2^ against ALL reflections. The weighted R-factor wR and goodness of fit S are based on F^2^, conventional R-factors R are based on F, with F set to zero for negative F^2^. The threshold expression of F^2^ > 2sigma(F^2^) is used only for calculating R-factors(gt) etc. and is not relevant to the choice of reflections for refinement. R-factors based on F^2^ are statistically about twice as large as those based on F, and R- factors based on ALL data will be even larger.

### Fractional atomic coordinates and isotropic or equivalent isotropic displacement parameters (Å^2^)

**Table:**

	*x*	*y*	*z*	*U*_iso_*/*U*_eq_	
C1	0.6358 (4)	−0.0602 (4)	0.1174 (7)	0.0299 (16)	
C2	0.6886 (4)	−0.1295 (4)	0.1037 (7)	0.0362 (17)	
C3	0.7615 (4)	−0.1247 (5)	0.0316 (7)	0.0397 (18)	
H3	0.7938	−0.1719	0.0252	0.048*	
C4	0.7876 (5)	−0.0496 (4)	−0.0323 (8)	0.0416 (18)	
C5	0.7398 (5)	0.0202 (5)	−0.0212 (8)	0.0445 (19)	
H5	0.7569	0.0706	−0.0629	0.053*	
C6	0.6647 (4)	0.0157 (4)	0.0534 (7)	0.0319 (16)	
C7	0.6152 (4)	0.0890 (4)	0.0576 (7)	0.0356 (17)	
H7	0.6320	0.1342	−0.0009	0.043*	
C8	0.5034 (5)	0.1751 (4)	0.1100 (7)	0.0389 (17)	
H8A	0.4493	0.1598	0.0813	0.047*	
H8B	0.5265	0.2066	0.0265	0.047*	
C9	0.5000	0.2309 (5)	0.2500	0.038 (2)	
C10	0.4250 (5)	0.2865 (5)	0.2380 (10)	0.062 (2)	
H10A	0.3778	0.2522	0.2412	0.093*	
H10B	0.4263	0.3168	0.1438	0.093*	
H10C	0.4240	0.3252	0.3214	0.093*	
I1	0.65256 (3)	−0.24569 (3)	0.19386 (5)	0.04569 (18)	
I2	0.89983 (4)	−0.04176 (4)	−0.13554 (7)	0.0658 (2)	
Ni1	0.5000	0.01657 (7)	0.2500	0.0310 (3)	
N1	0.5506 (3)	0.0986 (3)	0.1332 (6)	0.0339 (13)	
O1	0.5668 (3)	−0.0689 (3)	0.1835 (5)	0.0363 (11)	

### Atomic displacement parameters (Å^2^)

**Table:**

	*U*^11^	*U*^22^	*U*^33^	*U*^12^	*U*^13^	*U*^23^
C1	0.031 (4)	0.027 (4)	0.032 (4)	0.006 (3)	0.002 (3)	0.000 (3)
C2	0.041 (4)	0.028 (4)	0.039 (4)	0.007 (4)	0.002 (3)	0.002 (3)
C3	0.042 (5)	0.035 (4)	0.042 (4)	0.009 (4)	−0.008 (4)	−0.006 (3)
C4	0.044 (5)	0.032 (4)	0.049 (4)	0.003 (4)	0.006 (4)	−0.005 (3)
C5	0.053 (5)	0.042 (5)	0.039 (4)	−0.006 (4)	0.005 (4)	0.001 (3)
C6	0.035 (4)	0.023 (4)	0.038 (4)	−0.002 (3)	−0.001 (3)	0.007 (3)
C7	0.043 (5)	0.023 (4)	0.041 (4)	−0.002 (4)	0.004 (4)	0.007 (3)
C8	0.046 (5)	0.026 (4)	0.045 (4)	0.005 (4)	0.000 (4)	0.006 (3)
C9	0.054 (7)	0.012 (5)	0.049 (6)	0.000	0.006 (5)	0.000
C10	0.068 (6)	0.053 (5)	0.066 (5)	0.019 (5)	0.009 (5)	−0.004 (4)
I1	0.0588 (4)	0.0285 (3)	0.0498 (3)	0.0072 (3)	0.0027 (3)	0.0056 (2)
I2	0.0448 (4)	0.0558 (4)	0.0967 (5)	−0.0031 (3)	0.0273 (3)	−0.0059 (3)
Ni1	0.0323 (7)	0.0229 (7)	0.0378 (7)	0.000	0.0032 (6)	0.000
N1	0.038 (4)	0.021 (3)	0.043 (3)	0.008 (3)	0.005 (3)	−0.001 (2)
O1	0.035 (3)	0.025 (2)	0.049 (3)	0.005 (2)	0.013 (2)	0.002 (2)

### Geometric parameters (Å, º)

**Table:**

C1—O1	1.297 (8)	C8—N1	1.469 (8)
C1—C2	1.422 (9)	C8—C9	1.521 (8)
C1—C6	1.423 (8)	C8—H8A	0.9700
C2—C3	1.374 (9)	C8—H8B	0.9700
C2—I1	2.108 (7)	C9—C8^i^	1.521 (8)
C3—C4	1.396 (9)	C9—C10^i^	1.540 (9)
C3—H3	0.9300	C9—C10	1.540 (9)
C4—C5	1.376 (10)	C10—H10A	0.9600
C4—I2	2.085 (8)	C10—H10B	0.9600
C5—C6	1.417 (10)	C10—H10C	0.9600
C5—H5	0.9300	Ni1—O1^i^	1.858 (4)
C6—C7	1.434 (9)	Ni1—O1	1.858 (4)
C7—N1	1.275 (8)	Ni1—N1	1.868 (5)
C7—H7	0.9300	Ni1—N1^i^	1.868 (5)
			
O1—C1—C2	120.3 (6)	C9—C8—H8B	108.9
O1—C1—C6	124.7 (6)	H8A—C8—H8B	107.7
C2—C1—C6	115.0 (6)	C8^i^—C9—C8	108.2 (7)
C3—C2—C1	123.0 (6)	C8^i^—C9—C10^i^	108.3 (4)
C3—C2—I1	118.4 (5)	C8—C9—C10^i^	111.3 (4)
C1—C2—I1	118.6 (5)	C8^i^—C9—C10	111.3 (4)
C2—C3—C4	120.7 (7)	C8—C9—C10	108.3 (4)
C2—C3—H3	119.7	C10^i^—C9—C10	109.4 (9)
C4—C3—H3	119.7	C9—C10—H10A	109.5
C5—C4—C3	119.4 (7)	C9—C10—H10B	109.5
C5—C4—I2	120.2 (5)	H10A—C10—H10B	109.5
C3—C4—I2	120.4 (5)	C9—C10—H10C	109.5
C4—C5—C6	120.3 (7)	H10A—C10—H10C	109.5
C4—C5—H5	119.9	H10B—C10—H10C	109.5
C6—C5—H5	119.9	O1^i^—Ni1—O1	85.2 (3)
C5—C6—C1	121.8 (6)	O1^i^—Ni1—N1	163.9 (2)
C5—C6—C7	118.6 (6)	O1—Ni1—N1	94.2 (2)
C1—C6—C7	119.5 (6)	O1^i^—Ni1—N1^i^	94.2 (2)
N1—C7—C6	126.7 (6)	O1—Ni1—N1^i^	163.9 (2)
N1—C7—H7	116.6	N1—Ni1—N1^i^	90.7 (3)
C6—C7—H7	116.6	C7—N1—C8	118.7 (6)
N1—C8—C9	113.3 (5)	C7—N1—Ni1	125.7 (5)
N1—C8—H8A	108.9	C8—N1—Ni1	114.8 (4)
C9—C8—H8A	108.9	C1—O1—Ni1	126.5 (4)
N1—C8—H8B	108.9		
			
O1—C1—C2—C3	178.3 (6)	N1—C8—C9—C8^i^	−36.1 (4)
C6—C1—C2—C3	−1.2 (10)	N1—C8—C9—C10^i^	82.8 (7)
O1—C1—C2—I1	−0.4 (8)	N1—C8—C9—C10	−157.0 (6)
C6—C1—C2—I1	−179.9 (5)	C6—C7—N1—C8	−173.4 (6)
C1—C2—C3—C4	0.3 (11)	C6—C7—N1—Ni1	−4.0 (10)
I1—C2—C3—C4	178.9 (5)	C9—C8—N1—C7	−114.5 (7)
C2—C3—C4—C5	0.6 (11)	C9—C8—N1—Ni1	75.0 (6)
C2—C3—C4—I2	178.1 (5)	O1^i^—Ni1—N1—C7	−95.4 (9)
C3—C4—C5—C6	−0.4 (11)	O1—Ni1—N1—C7	−8.0 (6)
I2—C4—C5—C6	−177.9 (5)	N1^i^—Ni1—N1—C7	156.7 (7)
C4—C5—C6—C1	−0.7 (11)	O1^i^—Ni1—N1—C8	74.4 (9)
C4—C5—C6—C7	−177.7 (6)	O1—Ni1—N1—C8	161.7 (4)
O1—C1—C6—C5	−178.1 (6)	N1^i^—Ni1—N1—C8	−33.6 (3)
C2—C1—C6—C5	1.4 (9)	C2—C1—O1—Ni1	165.6 (5)
O1—C1—C6—C7	−1.1 (10)	C6—C1—O1—Ni1	−14.9 (9)
C2—C1—C6—C7	178.4 (6)	O1^i^—Ni1—O1—C1	−178.8 (6)
C5—C6—C7—N1	−171.8 (7)	N1—Ni1—O1—C1	17.2 (6)
C1—C6—C7—N1	11.1 (11)	N1^i^—Ni1—O1—C1	−90.2 (9)

Symmetry code: (i) −*x*+1, *y*, −*z*+1/2.
